# Single Incision Laparoscopic Colectomy: Technical Aspects, Feasibility, and Expected Benefits

**DOI:** 10.1155/2010/913216

**Published:** 2010-05-30

**Authors:** F. Leblanc, B. J. Champagne, K. M. Augestad, S. L. Stein, E. Marderstein, H. L. Reynolds, C. P. Delaney

**Affiliations:** Division of Colorectal Surgery, University Hospitals Case Medical Center, 11100 Euclid Avenue, Cleveland, OH 44106-5047, USA

## Abstract

*Background*. This paper studied technical aspects and feasibility of single incision laparoscopic colectomy (SILC). *Methods*. Bibliographic search was carried out up to October 2009 including original articles, case reports, and technical notes. Assessed criteria were techniques, operative time, scar length, conversion, complications, and hospitalization duration. *Results*. The review analyzed seventeen SILCs by seven surgical teams. A single port system was used by four teams. No team used the same laparoscope. Two teams used two laparoscopes. All teams used curved instruments. SILC time was 116 ± 34 minutes. Final scar was longer than port incision (31 ± 7 versus 24 ± 8 mm; *P* = .036). No conversion was reported. The only complication was a bacteremia. Hospitalization was 5 ± 2 days. *Conclusion*. SILC is feasible. A single incision around the umbilical scar represents cosmetic progress. Comparative studies are needed to assess potential abdominal wall and recovery benefits to justify the increased cost of SILC.

## 1. Introduction

Most surgeons are now convinced of the benefits of the laparoscopic approach in colorectal surgery [1–4]. While the advantages of laparoscopic surgery include shorter postoperative stay, early return of bowel function, and decreased complications, the disadvantages of multiport laparoscopic colectomy technique, include three to five port sites in the abdomen an additional incision to remove the specimen. 

The excitement to develop new techniques, to improve cosmesis and hasten recovery, has given rise to the natural orifice transluminal endoscopic surgery (NOTES), and more recently to single incision laparoscopic surgery (SILS). The initial applications of SILS in gastrointestinal surgery were appendicectomy and cholecystectomy [14, 15]. The guiding principle is operating through a single transumbilical incision, and removing the colonic specimen by the same small incision [5, 6, 9]. Compared to classic laparoscopic colectomy, the potential advantages of the SILS are believed to be reduction in cutaneous and parietal trauma, decreased postoperative pain, improved cosmesis, and shorter recovery, hopefully without additional cost [12, 13]. 

The aim of this paper was to analyze the current literature on single incision laparoscopic colectomy (SILC) including safety, techniques and feasibility and to assess the potential benefits of this new technique. 

## 2. Methods

### 2.1. Articles Identification

Databases consulted to carry out the search for relevant articles were Medline, The Cochrane Database of Systematic Reviews and Controlled Trials Register, The York Centre for Reviews and Dissemination, http://clinicaltrials.gov/, and The National Research Register of the National Health Service. Relevant articles and websites related to the study topic were also reviewed. The search was conducted without language restriction from January 2005 to October 2009 (inclusive). The search keywords used were triport system, single-port laparoscopic surgery, single incision laparoscopic surgery (SILS), embryonic natural orifice transumbilical endoscopic surgery (E-NOTES), and laparoendoscopic single-site surgery (LESS).

### 2.2. Articles Selection

Inclusion criteria were original articles, case reports, and technical notes, adult human patients, colorectal surgery, and robotic-assistance or not, without restriction of operative indication, disease, or surgical procedure. Exclusion criteria were editorials, congress abstracts, letters, experimental studies (cadaver, animal), minilaparotomy, multiport and hand-assisted laparoscopic surgery, natural orifice transluminal endoscopic surgery (NOTES), and transanal endoscopic microsurgery (TEM).

### 2.3. Studies Abstraction Synthesis

Data were extracted by the same surgeon reviewer (F Leblanc) experienced in laparoscopic colorectal surgery. Expected assessment criteria were: preoperative bowel preparation, surgical material, operative technique, operative time, conversion, incision length, complications, and hospitalization duration. Parametric data were expressed as mean ± SD and compared using the Mann Whitney *U* test. *P* < .05 was considered as significant.

## 3. Results

### 3.1. Studies Selection and Characteristics

**Figure 1:**
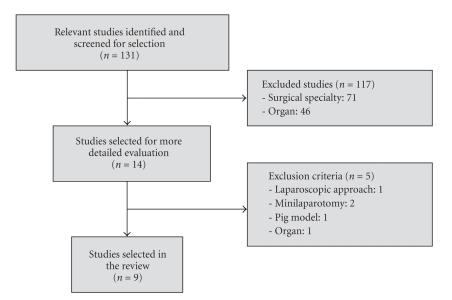
Single incision laparoscopic colorectal surgery: studies selection.

The primary search identified 131 potentially relevant studies (Figure 1). Adjusting to selected criteria, 122 studies were excluded. Nine studies, meeting all the inclusion criteria and representing the experience of 7 surgical teams, were analyzed. These included 5 technical notes, 2 case series, and 2 original articles, permitting the analysis of 17 cases of single incision laparoscopic colectomies: 11 right and 6 left colectomies to treat benign (*n* = 3) and malignant or potential malignant (*n* = 12) diseases (Table 1). 

### 3.2. Bowel Preparation

A preoperative bowel preparation was reported in 3 studies and not specified in 6 studies (Tables 3 and 4). A bowel preparation was performed in a single study for a sigmoid colectomy during which a preoperative sigmoidoscopy was performed (Table 4). 

### 3.3. Surgical Material and Operative Techniques

**Figure 2:**
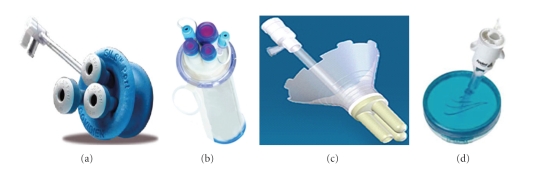
Single port systems used in the studies selected. (a) SILS Port (Covidien, Norwalk, Connecticut, USA); (b) ASC Triport (Advanced Surgical Concepts, Wicklow, Ireland); (c) Uni-X (Pnavel Systems, Morganville, New Jersey, USA); (d) GelPort (Applied Medical, Rancho Santa Margarita, California, USA).

Four surgical teams used a single port system, and three teams used trocars inserted directly through the skin incision (Table 2, Figure 2). One surgical team initially used trocars (2 first cases) then modified the technique to a single port system because of a pneumoperitoneum leak around trocars (Ostrowitz). A variety of laparoscopes sizes, tips, and angulations were used. Three teams used a curved laparoscope (angular or flexible), four teams selected a 30° laparoscope, and two teams used two different diameter laparoscopes interchangeably (Table 2). All teams selected curved laparoscopic instruments. One team used robotic-assistance.

To insert the port system, the skin incision measured 24 ± 8 mm long in average (*n* = 16) (Table 5). Mesentery and colon were exposed using graspers (*n* = 17), transparietal stitches (*n* = 5), and a sigmoidoscope with a magnetic anvil (*n* = 1) (Tables 3 and 4). Both medial to lateral and lateral to medial approaches were used. The ligation of the vessels was performed electrothermally in seven studies and tied or stapled in two studies. An ileotransverse anastomosis was stapled extracorporeally in four studies and intracorporeally in one study (Table 3).

### 3.4. Operative Results

Surgery was performed for a variety of benign and malignant diseases (Table 1). Mean final scar length was 31 ± 7 mm (*n* = 11) and was significantly higher than the initial skin incision (*P* = .036) (Table 5). Mean SILC time was 116 ± 34 minutes (*n* = 16). No conversion to straight, hand-assisted laparoscopy or laparotomy was reported. Mean specimen length was 30 ± 10 cm (*n* = 6). Proximal and distal colonic margins were described in two cases for malignancy and were noted to be >10 cm [6, 8]. Mean number of removed lymph nodes for malignant and potential malignant diseases was 17 ± 8 (*n* = 9). No intraoperative complication and only one postoperative complication, an enterobacter bacteremia on a dialyzed patient, were reported. Mean hospitalization duration was 5 ± 2 days (*n* = 9). 

## 4. Discussion

To date, only single case reports and small case series were available evaluating the success of Single Incision Laparoscopic Colectomy. Although multiple names have been used, Single Incision Laparoscopic Surgery appears to be the most accurate term to describe the variety of techniques utilized. This paper of nine articles analyzes the technical aspects and operative results of SILS for colectomy. It combines data from seven different laparoscopic surgery teams. The data reviewed in this study suggest the safety and feasibility of SILC. The mean operative time in our analysis was 116 minutes. This compares favorably with mean published operative time of 178 minutes for a multiport laparoscopic colectomy in a multicenter trial of 872 patients [16]. That suggests that SILC may be as fast as multiport laparoscopic colectomy, albeit in selected cases performed by selected surgeons, with reporting bias for successful cases using a new technique. 

The number of examined nodes and the colonic specimen length to treat malignant or potential malignant tumors appears oncologically satisfactory. Nonetheless, data were inadequate about the colonic margins and the surgical quality of colonic resection to validate the oncologic feasibility of SILC. 

Potential advantages of SILC over multiport laparoscopic colectomy include a single small skin incision. The length of the skin incision is dictated in part by specimen size. Extraction difficulties may be encountered for large colonic tumors, or obese patients with thickened mesentery, omentum, or deep abdominal wall. In addition, when the colon is full of stool, it may be difficult to extract. A bowel preparation may reduce the colonic diameter and incision length in these cases. In this paper, the size of the final skin incision was significantly longer than the initial incision, suggesting that analysis of the cosmetic benefits of the SILC should be based on final rather than initial scar length and device diameter. A better indicator of postoperative cosmetic result might be a blinded assessment of the abdomen after recovery from SILC compared with the abdominal incisions after traditional laparoscopic colectomy.

Theoretically, a single midline fascial incision minimizes trauma to the abdominal muscles, epigastric arteries, and parietal nerves created by placement of several trocars, potentially reducing postoperative wall pain. Data were not available to assess any analgesic advantage of SILC. No study included specifics on postoperative pain scores or analgesic requirements.

Furthermore, a single incision may decrease postoperative hernia rate. Published data on port-site hernias after multiport laparoscopic surgery and intraoperative closure are low, with an estimate of 0.14% [17]. However, the data on extraction sites after laparoscopic colectomy demonstrated significantly higher rates. A prospective comparative study of 166 patients found a significantly higher rate hernia through the midline than other extractions sites (17% versus 0%; *P* < .0002) [18]. The larger, single transumbilical fascial incision may increase the midline hernia rate. However a study will be necessary. To maintain cosmesis, SILS uses a midline transumbilical fascial incision. Thus, the incidence of incisional hernia could increase with SILS even if this approach avoids peripheral port-site hernias. 

The length of stay did not appear to be decreased using SILS technology. The duration of hospitalization after a multiport laparoscopic colectomy is estimated at 5 days [3]. In our paper, the duration of hospitalization was also 5 days, not demonstrating any advantage of SILS on recovery. No data was available on return of bowel function. The cost of SILS is also an issue in the current health care climate. The use of trocars through a GelPort, multiple laparoscopes, curved instruments, and robotic-assistance makes it very difficult to demonstrate any cost benefit for this approach in comparison with standard multiport laparoscopic surgery. Only an improvement in recovery, hospital stay or complications would make SILS cost effective.

SILS presents several disadvantages compared to multiport laparoscopic surgery. Externally, the handling of both straight instruments in parallel with the laparoscope through a small single incision decreases the freedom of motion for the surgeon and complicates the holding of the laparoscope for the assistant. To reduce the lines and cords that clutter the operative table, a small diameter laparoscope with an angular tip and an incorporated light source were used by several teams [5, 6, 9]. One surgical team proposed also to use three trocars through a gelport to increase the freedom of motion [10]. Inside the peritoneal cavity, lack of instrument triangulation increases the complexity of colonic exposure and dissection. To improve view and dissection, a 30° laparoscope and articulating or curved graspers and/or scissors were used by some authors. In our experience, we have found the best results and least technical difficulty with straight instruments. The use of trocars without a device exposes the surgeon to the loss of the pneumoperitoneum as was demonstrated in one study [13]. In case of intraoperative difficulties, SILS always offers the possibility to rapidly convert to multiport laparoscopic surgery, permitting the advantages of laparoscopic surgery to be preserved.

Lastly, SILS presents challenge for teaching laparoscopy. The mechanics of the operation are best suited to a single operator and this may hinder the training of surgeons in SILS. The potential difficulty in training residents and surgeons in this advanced technique needs to be addressed. Despite published benefits of minimally invasive colectomy, a prolonged learning curve had led to low adoption rate. SILS with its new technical and training challenges may not be accessible to most surgeons and most patients in the near future. 

## 5. Conclusion

For experienced laparoscopic colorectal surgeons, single incision laparoscopic colectomy is safe, feasible although technically more difficult than straight multiport laparoscopic colectomy. SILC may present cosmetic advantages in comparison to the multiport laparoscopic colectomy. Nevertheless, to determine its benefits, larger comparative studies to multiport laparoscopic colectomy with cost analysis, oncologic outcomes, and long-term follow-up will be necessary. 

## Figures and Tables

**Table 1:** Review of single incision laparoscopic colorectal surgery: included studies.

Study [ref]	Article	Cases (sex)	Age	BMI (kg/m^2^)	Indication	Colectomy
Remzi et al. [5]	Original	1 (F)	67	35	Polyp	Right
Bucher et al. [6]	Case	1 (ns)	81	ns	Polyp	Right
Bucher et al. [7]	TN	1 (F)	34	22	EM	Sigmoid
Bucher et al. [8]	TN	1 (M)	56	26	Polyp	Left
Leroy et al. [9]	TN	1 (F)	40	21	DV	Sigmoid
Merchant and Lin [10]	TN	ns	ns	ns	ns	Right
Brunner et al. [11]	TN	2 (F)	56	ns	DV	2 sigmoid
			42		EM	
Rieger and Lam [12]	Series	7 (6M-1F)	(60–83)	(22–28)	4 cancers	6 right
					2 Polyps	1 left flexure
Ostrowitz et al. [13]	Original	3 (2M-1F)	(74–82)	ns	1 cancer	3 right
					2 villous	

TN: technical note; EM: endometriosis; DV: diverticulitis; ns: not specified.

**Table 2:** Single incision laparoscopic colorectal surgery: material required.

Study [ref]	Port system		Laparoscope			Graspers/Scissors
	Single port (diameter, mm)	Trocars (diameter, mm)	Tip	Diameter (mm)	Degree	
Brunner et al. [11]	None	3 trocars (5, 5, 5)	Rigid/Straight	5	30°	AR–ST/ns

Remzi et al. [5]	Triport (5, 5, 5)	None	Flexible	5 (incorporated light source)	ns	Curved/Curved

Rieger and Lam [12]	None	3 trocars (12, 5, 5)	ns/ns	10	30°	ST/AR

Merchant and Lin [10]	Gelport	3 trocars (10, 5, 5)	Rigid/Straight	5	30°	AR/ns

Bucher et al. [6–8]	None	2 trocars (12, 5)	Rigid/Angular	10 (6 mm working channel)	ns	AR/ST
			Rigid/Straight	5	30°	

Leroy et al. [9]	Triport (10, 5, 5)	None	Rigid/Angular	10	0°	AR/AR
			Rigid/ns	3	0°	

Ostrowitz et al. [13]	Triport (12, 8, 8)	3 trocars (12, 8, 8)	ns/ns	12	ns	AR^#^/ns
	Third case	Two first cases				

TN: technical note; EM: endometriosis; DV: diverticulitis; ns: not specified.

**Table 3:** Techniques step by step of single incision laparoscopic right colectomy.

Study [ref]	Bowel preparation	Exposure	Mesenteric dissection	Vessels ligation	Proximal section	Distal section	Anastomosis
Remzi et al. [5]	ns	Grasping	Lateral to medial	Electrothermal	Extracorporeal	Extracorporeal	Extracorporeal
			Scissors		Stapled	Stapled	Stapled

Merchant and Lin [10]	ns	Grasping	Medial to lateral	Stapled	Intracorporeal	Intracorporeal	Intracorporeal
					Stapled	Stapled	Stapled

Bucher et al. [6]	ns	Grasping	Medial to lateral	Knotting	ns	ns	Extracorporeal
		Transparietal stitches	Scissors/Hook/Ultrasound				Stapled

Rieger and Lam [12]	None*	Grasping	Lateral to medial	Electrothermal	ns	ns	Extracorporeal
			Scissors	Knotting			Stapled

Ostrowitz et al. [13]	ns	Grasping^#^	Medial to lateral	Electrothermal	Extracorporeal	Extracorporeal	Extracorporeal
			Hook^#^		Stapled	Stapled	Stapled

^#^Robotic-assistance; *Preoperative coloscopic marking of the tumor; ns: not specified.

**Table 4:** Techniques step by step of single incision laparoscopic sigmoid and left colectomies.

Study [ref]	Bowel preparation	Exposure	Mesenteric dissection	Vessels ligation	Proximal section	Distal section	Anastomosis
Brunner et al. [11]	ns	Grasping	Medial to lateral	Electrothermal	Extracorporeal	Intracorporeal	Intracorporeal
		Transparietal stitches	Electrothermal		ns	Stapled	Stapled

Bucher et al. [7, 8]	ns^#^	Grasping	Medial to lateral	Electrothermal	ns	Intracorporeal	Intracorporeal
		Transparietal stitches	Scissors/Hook			Stapled	Stapled

	fiber-free diet	Grasping	Lateral to medial	Electrothermal	Intracorporeal	Intracorporeal	Intracorporeal
Leroy et al. [9]	PEG	Sigmoidoscopy	Electrothermal		Stapled	Stapled	Stapled
	enema per ano	IL magnetic anvil					

Rieger* and Lam [12]	None^#^	Grasping	Lateral to medial	Electrothermal	ns	ns	Extracorporeal
			Scissors				Manual

^#^Preoperative coloscopic marking of the tumor; IL: intraluminal; *Left flexure colectomy; ns: not specified.

**Table 5:** Review of single incision laparoscopic colectomy: results.

Study [ref]	Colectomy	Skin Incision length		Time (min)	Specimen (cm)	Lymph nodes	Stay (day)
		Initial (mm)	Final (mm)				
Remzi et al. [5]	Right	35	35	115	ns	ns	4

Leroy et al. [9]	Sigmoid	20	20	90	40	ns	4

Brunner et al. [11]	Sigmoid	20	ns	110	22	ns	7
	Sigmoid	20	ns	180	18	ns	6

	Right	ns	30	158	38	33	ns
Bucher et al. [6–8]	Sigmoid	20	ns	125	23	14	2
	left	20	ns	ns	39	ns	ns

	Right	40	40	132	ns	22	4
Ostrowitz et al. [13]	Right	40	ns	158	ns	ns	3
	Right	2.5	ns	166	ns	ns	4

	Right	25	35	100	ns	10	ns
	Right	25	35	90	ns	26	ns
	Right	25	25	75	ns	16	ns
Rieger and Lam [12]	Right	25	45	115	ns	10	11
	Right	25	30	80	ns	7	ns
	Right	25	25	88	ns	21	ns
	LF	25	25	75	ns	12	ns

TN: technical note; EM: endometriosis; DV: diverticulitis; ns: not specified.
